# Selective pharmacological blockade of the 5-HT7 receptor attenuates light and 8-OH-DPAT induced phase shifts of mouse circadian wheel running activity

**DOI:** 10.3389/fnbeh.2014.00453

**Published:** 2015-01-15

**Authors:** Jonathan Shelton, Sujin Yun, Susan Losee Olson, Fred Turek, Pascal Bonaventure, Curt Dvorak, Timothy Lovenberg, Christine Dugovic

**Affiliations:** ^1^Neuroscience, Janssen Research and Development, LLCSan Diego, CA, USA; ^2^Department of Neurobiology, Center for Sleep and Circadian Biology, Northwestern UniversityEvanston, IL, USA

**Keywords:** circadian rhythms, 5-HT7 receptor, phase shift, photic, non-photic, depression, mood, serotonin

## Abstract

Recent reports have illustrated a reciprocal relationship between circadian rhythm disruption and mood disorders. The 5-HT7 receptor may provide a crucial link between the two sides of this equation since the receptor plays a critical role in sleep, depression, and circadian rhythm regulation. To further define the role of the 5-HT7 receptor as a potential pharmacotherapy to correct circadian rhythm disruptions, the current study utilized the selective 5-HT7 antagonist JNJ-18038683 (10 mg/kg) in three different circadian paradigms. While JNJ-18038683 was ineffective at phase shifting the onset of wheel running activity in mice when administered at different circadian time (CT) points across the circadian cycle, pretreatment with JNJ-18038683 blocked non-photic phase advance (CT6) induced by the 5-HT1A/7 receptor agonist 8-OH-DPAT (3 mg/kg). Since light induced phase shifts in mammals are partially mediated via the modulation of the serotonergic system, we determined if JNJ-18038683 altered phase shifts induced by a light pulse at times known to phase delay (CT15) or advance (CT22) wheel running activity in free running mice. Light exposure resulted in a robust shift in the onset of activity in vehicle treated animals at both times tested. Administration of JNJ-18038683 significantly attenuated the light induced phase delay and completely blocked the phase advance. The current study demonstrates that pharmacological blockade of the 5-HT7 receptor by JNJ-18038683 blunts both non-photic and photic phase shifts of circadian wheel running activity in mice. These findings highlight the importance of the 5-HT7 receptor in modulating circadian rhythms. Due to the opposite modulating effects of light resetting between diurnal and nocturnal species, pharmacotherapy targeting the 5-HT7 receptor in conjunction with bright light therapy may prove therapeutically beneficial by correcting the desynchronization of internal rhythms observed in depressed individuals.

## Introduction

Circadian rhythms are governed by a variety of environmental inputs including light, food, social interaction, and pharmacological agents (Lall et al., 2012; Bloch et al., 2013; Patton and Mistlberger, 2013; Pendergast and Yamazaki, 2014). Of these, light is the most powerful of the synchronizing agents (Klein et al., 1991) and exerts its influence on circadian rhythms by first exciting a subset of retinal ganglion cells that subsequently activate neurons within the master circadian clock located in the suprachiasmatic nucleus (SCN) via the retinohypothalamic tract (RHT; Moore and Lenn, 1972; Gooley et al., 2001; Hattar et al., 2002; Panda et al., 2002; Ruby et al., 2002). In addition to photic input, serotoninergic pathways can also exert non-photic influence over the synchronization of circadian rhythms either by direct projections from raphe nucleus onto the SCN or indirect via the intergeniculate leaflet onto the SCN (Moga and Moore, 1997; Pickard and Rea, 1997; Ciarleglio et al., 2011). Working in accordance with each other, the photic and non-photic pathways help ensure proper synchronization of circadian rhythms by responding to a variety of changes in the environment. Recent studies have demonstrated that the misalignment or the inability to adjust to such derivations in the environment give rise to mood disorders in humans (Sprouse, 2004; Grandin et al., 2006; Murray and Harvey, 2010; Salvadore et al., 2010).

Utilizing a “clock in a dish” model, a role for the 5-HT7 receptor in the modulation of the non-photic regulation of circadian rhythms was noted for the first time when the receptor was initially cloned (Lovenberg et al., 1993). This method demonstrated that the phase advance in neuronal activity of the SCN following administration of the 5-HT1A/7 receptor agonist 8-OH-DPAT was attenuated by the 5-HT7/2 receptor antagonist ritanserin, but not by pindolol, a 5-HT1A/1B receptor antagonist (Lovenberg et al., 1993). This finding was later confirmed with the more selective 5-HT7 receptor antagonist SB-269970 (Sprouse et al., 2004). *In vivo* studies later reported the translational aspects of this cell model by demonstrating that phase advances in wheel running activity induced by 8-OH-DPAT were blocked following the administration of the 5-HT7 receptor antagonist DR-4004 in hamsters and absent in the 5-HT7 receptor knockout (KO) mouse (Ying and Rusak, 1997; Ehlen et al., 2001; Horikawa and Shibata, 2004; Gardani and Biello, 2008; Horikawa et al., 2013). In addition to its effects on the non-photic regulation of circadian rhythms, there is evidence that the 5-HT7 receptor may also influence photic regulation of circadian rhythms either by altering the sensitivity to light or modulating the release of serotonin (Ying and Rusak, 1997; Smith et al., 2001).

In addition to regulating circadian rhythms, the 5-HT7 receptor has been studied extensively for its role in depression. Initial investigation noted that 5-HT7 receptor KO mice exhibited an antidepressant-like phenotype in models of depression such as the tail suspension and forced swim tests (Guscott et al., 2005; Hedlund et al., 2005). Comparable antidepressant-like effects were found in these behavioral tests with the selective 5-HT7 receptor antagonist SB-269970 (Wesołowska et al., 2006; Sarkisyan et al., 2010). While many studies have utilized SB-269970 as a tool compound to investigate a role for the 5-HT7 receptor in various physiologic systems and pathological states, its utility is hampered due to a short half-life and poor drug-like properties (Hagan et al., 2000). Our in-house efforts yielded JNJ-18038683, a selective 5-HT7 receptor antagonist that exhibits better pharmacokinetic properties than SB-269970. Pre-clinical and clinical evaluation of the compound demonstrated that JNJ-18038683 was efficacious in the mouse tail suspension test and also enhanced serotonin transmission, antidepressant-like properties, and REM sleep suppression induced by the selective serotonin reuptake inhibitor (SSRI) citalopram in rats (Bonaventure et al., 2012). The effects of JNJ-18038683 on REM sleep translated from rodents to humans whereas the antidepressant efficacy needed to be further assessed (Bonaventure et al., 2012). Interestingly, systemic administration of the selective 5-HT7 receptor agonist LP-211 significantly increased the time spent awake while the direct infusion of this compound into dorsal raphe nucleus, locus coeruleus, basal forebrain, or laterodorsal tegmental nucleus resulted in decreased duration of REM sleep (Monti et al., 2008, 2014; Monti and Jantos, 2014). Similar REM sleep suppressive effects were observed when another selective 5-HT7 receptor agonist LP-44 was injected directly into the dorsal raphe nucleus (Monti et al., 2008).

Given the association of the 5-HT7 receptor with mood and circadian rhythms, pharmacological manipulation of this receptor may provide a critical insight into the therapeutic link between depression and circadian disruption. Therefore, the current study was designed to examine a role for the 5-HT7 receptor in circadian rhythm regulation by administering the selective 5-HT7 receptor antagonist JNJ-18038683 in both photic and non-photic circadian paradigms. To determine if JNJ-18038683 exerts direct phase resetting properties, a phase response curve was generated by administering the compound to mice at select times throughout the circadian cycle. Second, mice were administered JNJ-18038683 to determine if the compound alters the non-photic phase shift of wheel running activity induced by 8-OH-DPAT. Finally, JNJ-18038683 was administered prior to a light pulse that occurred at times known to delay (circadian time (CT) 15) or advance (CT 22) the onset of wheel running activity (Daan and Pittendrigh, 1976; Takahashi et al., 1984) in mice to determine the effects of acute pharmacological blockade of the 5-HT7 receptor on photic induced phase shifts. Results of these studies demonstrate that the 5-HT7 receptor influences both photic and non-photic aspects of circadian regulation and therefore may provide a therapeutic avenue to alleviate circadian disruptions associated with depression.

## Methods

### Animals

Studies conducted for the current investigation were performed in accordance with the policies and regulations of the respective IACUC committees at Northwestern University and Janssen Research and Development, L.L.C. For the experiments outlined for the following studies, male C57Bl/6 J mice (average weight ~30 grams) were purchased from Jackson Labs (Sacramento, CA) and allowed to acclimate for at least 2 weeks before being moved to environmental chambers that allowed for the ability to control lighting schedules and modified cages that contained a running wheel. Mice were allowed access to food and water ad libitum and maintained under a 12 h light/12 h dark schedule.

### Drugs

JNJ-18038683 (1-Benzyl-3-(4-chlorophenyl)-1,4,5,6,7,8-hexahy­dropyrazolo[3,4-d]azepine) was synthesized by medicinal chemistry group at Janssen Research and Development, L.L.C. as the citrate salt form. For formulations, 20% (w/v) hydroxypropyl-β-cyclodextrin was used to solubilize the compound and a correction factor of 1.5690 was applied to compensate for the salt form of JNJ-18038683. 8-OH-DPAT ((±)-8-Hydroxy-2-dipropylaminotetralin hydrobromide) (Tocris Biosciences) was formulated in saline. A correction factor of 1.32 was applied to compensate for the hydrobromide salt form of 8-OH-DPAT. Both compounds were injected in a volume of 10 mL/kg body mass. With each compound, the pH of the solution was adjusted to neutral before injection.

### Experimental design

A phase response curve for JNJ-18038683 (10 mg/kg, i.p.) was generated by administering the compound at various times across the circadian cycle (CT2, 6, 10, 14, 18, or 22) to separate groups of mice for each time point. For these studies, a single animal was used in more than one injection condition and at least 2 weeks were given between conditions to prevent carry-over, or interference effects from the previous trial.

To determine if JNJ-18038683 alters the phase advance of wheel running activity induced by the 5-HT1A/7 receptor agonist, 8-OH-DPAT, mice were first randomized into four separate groups. Animals then received an injection of JNJ-18038683 (3 mg/kg, i.p.) or vehicle. Thirty minutes later, mice were further designated to receive 8-OH-DPAT (3 mg/kg, i.p.,) or corresponding vehicle at CT6 which had been previously reported to result in a phase advance of the onset of locomotor rhythm by 8-OH-DPAT (Horikawa and Shibata, 2004).

To investigate the role of the 5-HT7 receptor on photic induced phase shifts, JNJ-18036863 (10 mg/kg, s.c.) or vehicle was administered 30 min prior to an acute light pulse (200 lux, 30 min) that occurred at times that are known to produce a photic phase delay (CT 15) or advance (CT 22) of the onset of wheel running activity. As a control for the light pulse, mice were placed in the chamber but not exposed to light. For each treatment, separate groups of mice were used.

### Wheel running activity recording and analysis

Wheel running activity was recorded by a magnetic switch located on the wheel that transferred each revolution as an event to an IBM compatible computer in 5 min bins using ClockLab software. Following at least a one-week acclimation period to adjust to the running wheels and novel cage, mice were released into constant darkness to assess free-running conditions and the onset of activity was monitored. On designated experimental days, the onset of activity (designated by convention as CT 12) was calculated based on the free running phase advance of activity of the previous 7 days. All timing for injections and light exposure was predicated by the calculation of CT 12 for each animal. Wheel running activity was monitored for at least 1 week following treatment or corresponding control conditions for changes to the onset of activity. A phase shift was calculated using ClockLab software and defined as the difference in the onset of activity after treatment vs. onset of activity prior to treatment. Data are expressed as mean ± S.E.M. To determine if JNJ-18038683 resulted in a significant phase shift at various time points along the phase response curve, an unpaired *t*-test (Vehicle vs. JNJ-18038683 treated animals) was executed. A one factor, four-level ANOVA followed by *post hoc* analysis was performed for each of the remaining two studies to determine if the phase shift was significant with the four treatment groups (*p* < 0.05). For visualization, a phase advance is denoted as a positive value on the bar graph while a phase delay is graphed as a negative value.

## Results

### Phase response curve with JNJ-18038683

To determine the effects of the pharmacological blockade of the 5-HT7 receptor by JNJ-18038683 (10 mg/kg, i.p.), the compound was administered at various circadian times throughout the subjective circadian cycle (CT 2, 6, 10, 14, 18, 22) and the resulting phase shift was calculated post administration and compared to vehicle. At all times tested, JNJ-18038683 failed to elicit a phase shift as determined by unpaired *t*-test (Table 1). In a separate study, negative results were also obtained following the administration of a higher dose of JNJ-18038683 (20 mg/kg, i.p.) (data not shown).

**Table 1:** **Phase response curve following the administration of the 5-HT7 receptor antagonist JNJ-18038683 (10 mg/kg, i.p.) at various circadian times**.

	Phase shift (min.)	
CT	Vehicle	JNJ-18038683
2	0.0 ± 0.0	0.25 ± 0.25
6	0.0 ± 0.0	−0.15 ± 0.10
10	−0.20 ± 0.10	−0.29 ± 0.20
14	0.0 ± 0.0	−0.08 ± 0.08
18	0.0 ± 0.0	0.0 ± 0.0
22	0.0 ± 0.0	0.0 ± 0.0

### Non-photic regulation of circadian rhythms by the 5-HT7 receptor

**Figure 1:**
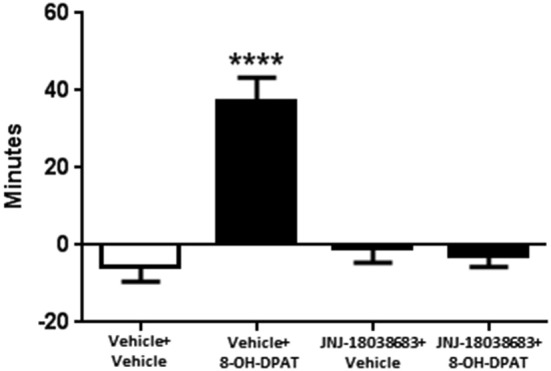
**Phase shifting effects of JNJ-18038683 (3 mg/kg. i.p.) and 8-OH-DPAT (3 mg/kg, i.p.) on the circadian rhythms of wheel running activity in mice**. Mice were randomly divided to receive one of four treatments: Vehicle + Vehicle (*n* = 14), Vehicle + 8-OH-DPAT (*n* = 21), JNJ-18038683 + Vehicle (*n* = 11), and JNJ-18038683 + 8-OH-DPAT (*n* = 16). After treatment, phase shifts of locomotor activity were calculated based upon the onset of activity following treatment compared to prior treatment. Results are expressed as mean ± S.E.M. and a one-way ANOVA followed by a Tukey’s *post hoc* test was performed to determine if a result was significant (****p < 0.0001 vs. Vehicle + Vehicle, JNJ-18038683 + Vehicle, and JNJ-18038683 + 8-OH-DPAT).

We then tested whether the 5-HT7 receptor antagonist JNJ-18038683 would attenuate the phase advance of wheel running locomotor activity elicited by the 5-HT1A/7 receptor agonist 8-OH-DPAT. Administration of the vehicle for JNJ-18038683 followed by 8-OH-DPAT resulted in a robust phase advance of the onset of locomotor activity during constant dark conditions (37.0 ± 6.3 min, *F*_(3,58)_ = 18.49 *p* < 0.0001, one-way ANOVA, Tukey’s *post hoc* analysis) when compared to the other three treatment groups (Vehicle + Vehicle: −5.4 ± 4.1 min, JNJ-18038683 + Vehicle −0.9 ± 3.6 min, JNJ-18038683 + 8-OH-DPAT: −2.7 ± 3.0 min) (Figure 1). Comparable to what had been measured during the generation of the phase response curve, the administration of JNJ-18038683 in conjunction with the vehicle for 8-OH-DPAT did not produce any phase shift at CT 6. The administration of the 5-HT7 receptor antagonist JNJ-18038683 completely blocked the phase advance produced by 8-OH-DPAT (Figure 1).

### Photic regulation of circadian rhythms by the 5-HT7 receptor

**Figure 2:**
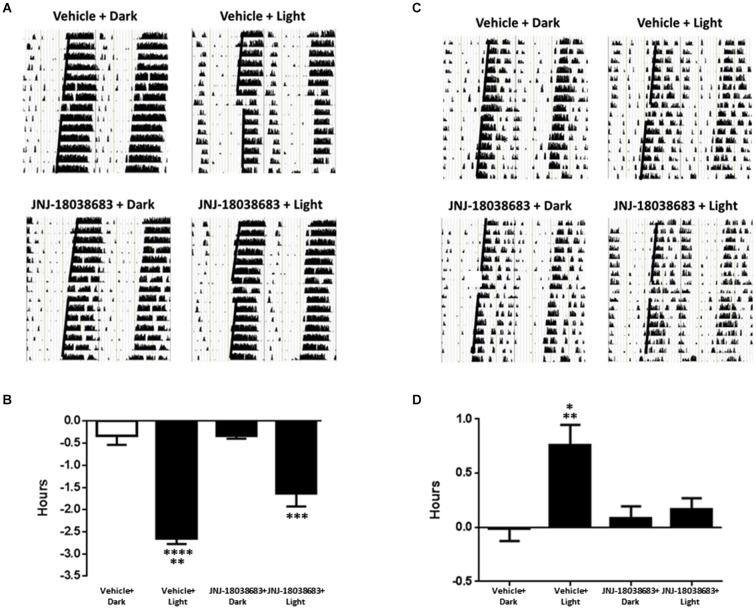
**The impact of the 5-HT7 receptor antagonist JNJ-18038683 (10 mg/kg, s.c.) on light induced phase shifts**. Representative actigrams and resulting phase shifts following administration of Vehicle/JNJ-18038683 or dark/light pulse on the circadian rhythms of wheel running activity in mice. Mice were randomly divided to receive one of four treatments: Vehicle + Dark (*n* = 5), Vehicle + Light Pulse (*n* = 6), JNJ-18038683 + Dark (*n* = 6), and JNJ-18038683 + Light Pulse (*n* = 5, CT 15; *n* = 6, CT 22). The light pulse was administered at times known to phase delay (CT15) **(A,B)** or advance (CT22) **(C,D)** the onset of wheel running activity in mice. After treatment, phase shifts of locomotor activity (onsets of activity indicated by bold black line) were calculated based upon the onset of activity following treatment compared to prior treatment. Results are expressed as mean ± S.E.M. and a one-way ANOVA followed by a Tukey’s *post hoc* test was performed to determine if a result was significant (Phase Delay—**p < 0.01 vs. JNJ-18038683 + Light, ***p < 0.001 vs. JNJ-18038683 + Dark, ****p < 0.0001 vs. Vehicle + Dark and JNJ-18038683 + Dark; Phase Advance—*p < 0.05 vs. JNJ-18038683 + Light, **p < 0.01 vs. Vehicle + Dark and JNJ-18038683 + Dark).

To determine the effects of pharmacological blockade of the 5-HT7 receptor on photic control of circadian rhythms, JNJ-18038683 was administered prior to a light pulse and the potential alterations in the onset of mouse wheel running activity was analyzed. In vehicle treated animals, light exposure resulted in a significant phase shift in the onset of activity at both times tested (CT 15: −2.65 ± 0.12 h: *F*_(3,18)_ = 42.37; CT22: 0.76 ± 0.19 h: *F*_(3,19)_ = 6.757 one way ANOVA, Tukey’s *post hoc* analysis) (Figures 2A–D). Administration of JNJ-18038683 attenuated the light-induced phase delay (−1.64 ± 0.29 h) (Figures 2C,D) while the resulting phase advance following light exposure at CT 22 was completely blocked by the compound (Figures 2A,B).

## Discussion

The present investigation examined the impact of the 5-HT7 receptor antagonist JNJ-18038683 on non-photic and photic regulation of the circadian rhythm of wheel running activity in mice. Results reported from these studies demonstrate that the compound did not exert direct non-photic or photic phase resetting properties by itself. However, JNJ-18038683 was able to block the non-photic phase advance of wheel running activity induced by the 5-HT1A/7 receptor agonist 8-OH-DPAT. In addition, JNJ-18038683 completely blocked the phase advance and significantly attenuated the phase delay of wheel running activity induced by a light pulse.

### Phase response curve of JNJ-18038683

To determine if JNJ-18038683 exerts direct non-photic or photic phase resetting effects in free running mice, the compound was administered at specified times across the circadian cycle. At all times tested, JNJ-18038683 failed to elicit a phase shift when compared to vehicle treated mice. The lack of effect with JNJ-18038683 on phase shifts in mice was similar to what has been reported for the 5-HT2/7 receptor antagonist, ritanserin and a more selective 5-HT7 receptor antagonist SB-269970 in hamsters and rats (Antle et al., 1998; Duncan et al., 2004; Westrich et al., 2013). While antagonists for the 5-HT7 receptor do not modulate non-photic regulation of circadian rhythms, agonists for this receptor (8-OH-DPAT and LP-211) are capable of shifting the onset of wheel running activity in constant conditions (Horikawa and Shibata, 2004; Adriani et al., 2012). In addition, fluoxetine that inhibits the reuptake of serotonin and therefore, universally activates the serotonergic system has been shown to induce non-photic phase shifts (Cuesta et al., 2008, 2009). Therefore, global activation of the serotonergic system or direct pharmacological activation of the 5-HT7 receptor may be needed to induce photic or non-photic phase shifts.

### Non-photic regulation of circadian rhythms by the 5-HT7 receptor

The second study was designed to determine if the acute pharmacological blockade of the 5-HT7 receptor with the selective antagonist JNJ-18038683 impacted the phase advance of wheel running activity induced by 8-OH-DPAT in mice. The timing of 8-OH-DPAT administration was based on previous studies that demonstrated a robust phase advance when the compound was injected at CT 6 (Horikawa and Shibata, 2004). When JNJ-18038683 was administered before 8-OH-DPAT in the current study, the phase advance induced by the agonist was completely blocked demonstrating the impact of the 5-HT7 receptor on non-photic phase resetting of circadian rhythms. Since 8-OH-DPAT is an agonist for both the 5-HT1 and 5-HT7 receptors, future studies will utilize selective agonists for the 5-HT7 receptor (i.e., LP-44 or LP-211) to further elucidate the role of the 5-HT7 receptor on non-photic regulation of circadian rhythms.

Earlier *in vivo* and *in vitro* studies from other labs have provided some insight into a mechanism by which the 5-HT7 receptor regulates non-photic control of circadian rhythms. During mid-subjective day, micro-injection of 8-OH-DPAT or 5-Carboxamidotryptamine (5-CT) into the dorsal raphe nucleus activates 5-HT7 receptors and subsequently phase advances the circadian rhythms of wheel running activity in hamsters (Mintz et al., 1997; Duncan et al., 2004; Duncan and Davis, 2005). These phase shifts were blocked by the administration of the selective 5-HT7 receptor antagonist SB-269970 (Duncan et al., 2004). Further investigation delineated this model to show that the activation of the 5-HT7 receptor inhibits glutamate release within the dorsal raphe nucleus resulting in the inhibition of the release of GABA from interneurons which subsequently relieves the inhibition on serotonergic neurons located within the dorsal or medial raphe nucleus (Glass et al., 2003; Harsing et al., 2004; Duncan and Congleton, 2010). The subsequent release of serotonin onto the SCN results in non-photic phase modulation (Duncan and Congleton, 2010). Therefore, administration of JNJ-18038683 may be acting on 5-HT7 receptors within the dorsal raphe nucleus to inhibit glutamate release and thus blocking the non-photic phase shifts induced by 8-OH-DPAT. However, further studies are needed to define specific pathways in which the 5-HT7 receptor modulates non-photic circadian re-setting since the receptor is localized not only within the dorsal/median raphe nucleus to modulate GABA release, but also within the primary circadian oscillator of the SCN and other brain regions known to influence circadian rhythms such as the thalamus that could potentially impact NPY release which has been shown to alter non-photic phase shifts (Mrosovsky, 1996; Belenky and Pickard, 2001; Neumaier et al., 2001; Bonaventure et al., 2002; Gamble et al., 2006; Matthys et al., 2011; Hughes and Piggins, 2012).

### Photic regulation of circadian rhythms by the 5-HT7 receptor

In addition to the non-photic influence of circadian rhythms, serotonin can also alter photic responsiveness of the SCN possibly by acting via the 5-HT7 receptor. Early studies associated the 5-HT7 receptor with changes in the sensitivity of light in SCN neurons. Administration of serotonin, 5-CT, or 8-OH-DPAT reduced the firing of SCN neurons located in the hamster following a light pulse (Ying and Rusak, 1997). This decrease in neuronal firing was subsequently reversed by applying ritanserin or clozapine which are known to antagonize the 5-HT7 receptor but not the 5-HT1A/B/D receptor antagonist, cyanopindolol or the 5-HT1A receptor antagonist WAY-100635 (Ying and Rusak, 1997). In subsequent studies, hypothalamic excitatory post synaptic currents evoked by optic nerve stimulation which mimics a light pulse were reduced when the 5-HT1A/B agonist TFMPP was added to the bath (Smith et al., 2001). The change in these glutamate dependent currents within the hypothalamus were significantly attenuated with ritanserin, thus further implicating a role for the 5-HT7 receptor in photic control of circadian rhythms by modulating glutamate transmission (Smith et al., 2001). The additional findings that TFMPP had little effect in this model in 5-HT1B receptor KO mice and was minimally attenuated with the 5-HT1A receptor antagonist WAY-100635, provide some indirect evidence that the role of the 5-HT7 receptor in modulating photic control of circadian rhythms may be in coordination with other serotonergic receptors and glutamatergic neurotransmission.

Additional studies have investigated a role for the 5-HT7 receptor in the photic control of circadian rhythms by generating a phase response curve of light in 5-HT7 receptor KO and corresponding WT mice (Gardani and Biello, 2008). Overall, the phase shifts induced by the light pulse were comparable in magnitude and direction between WT and 5-HT7 receptor KO mice except at CT 22 (Gardani and Biello, 2008). At this time point, the light pulse resulted in the requisite phase advance in WT mice but a phase delay in the 5-HT7 receptor KO mice (Gardani and Biello, 2008). Interestingly, while the current study measured a significant attenuation of the light induced phase delay at CT 15 with the administration of JNJ-18038683, there was no difference in WT or 5-HT7 receptor KO mouse when the light pulse was administered at CT 14 or 16 which replicated an earlier report (Sprouse et al., 2003). The difference in regards to the light induced phase shift at these earlier time points between the pharmacological and the genetic deletion of the receptor may be the result of compensatory mechanisms involved in the phase resetting after a light pulse at these earlier time points of the subjective dark phase in the 5-HT7 receptor KO mouse.

Since this is the first report of attenuation of phase shifts following light pulses following the acute pharmacological blockade of the 5-HT7 receptor, additional studies are needed to further investigate the mechanism by which the 5-HT7 receptor is modulating the photic control of circadian rhythms. Results from these studies will provide an explanation as to why pharmacological blockade of the 5-HT7 receptor resulted in diminished photic induced phase shifts as opposed to an enhancement given the finding that activation of the 5-HT7 receptor inhibits the RHT input to the SCN by decreasing glutamate (Ying and Rusak, 1997; Smith et al., 2001). In addition, since antagonists selective for the 5-HT7 receptor lack direct photic-like phase shifting effects by themselves, the actions of these 5-HT7 receptor antagonists are probably due to the coordination with other serotonin receptors including the 5-HT1A and 5-HT1B receptors (Ying and Rusak, 1997; Rea, 1998; Smith et al., 2001).

Similar to our findings that JNJ-18038683 diminished photic induced phase resetting, pharmacological agents that are known to activate the serotonin system such as SSRIs (fluoxetine, citalopram, fluvoxamine, and paroxetine) also reduce the phase shift induced by a light pulse in the hamster (Gannon and Millan, 2007). Interestingly, fluoxetine potentiates the light induced phase shift in the diurnal species *Arvicanthis ansorgei* (Cuesta et al., 2008). These contrasting effects between nocturnal and diurnal species may be explained by the differential regulation of brain concentrations of serotonin corresponding to arousal state and also may be due to the changes in expression of circadian controlled genes following a light pulse in nocturnal and diurnal animals (Faradji et al., 1983; Poncet et al., 1993; Rea et al., 1994; Weber et al., 1998; Cuesta et al., 2008). While the current study did not measure the changes in the expression of such genes in response to pharmacological manipulation of the 5-HT7 receptor, Westrich and colleagues reported that the *in vitro* period as measured by a luciferase reporter linked to the PER2 gene, can be shortened by the 5-HT7 receptor agonist AS-19 and subsequently blocked by the 5-HT7 receptor antagonist SB-269970 (Westrich et al., 2013).

Due to the possible potentiation of the effects of light in diurnal species such as humans, 5-HT7 receptor antagonists may provide an interesting adjunctive therapy for the antidepressant effects associated with bright light therapy. The idea of adjunctive therapy with a 5-HT7 receptor antagonist arose from earlier studies in which JNJ-18038683 or SB-269970 enhanced the antidepressant-like effects of an SSRI in pre-clinical studies (Bonaventure et al., 2007, 2012; Wesołowska et al., 2007). When JNJ-18038683 was administered in combination with citalopram, there was an enhancement of serotonin transmission, antidepressant-like behavior, and REM sleep suppression induced by the SSRI in rodents (Bonaventure et al., 2012). In another study, Westrich et al. demonstrated that the administration of escitalopram or the 5-HT7 receptor antagonist SB-269970 was ineffective at phase shifting wheel running activity in rats, however, the co-administration of escitalopram with SB-269970 resulted in phase delays in rodent wheel running (Westrich et al., 2013). In addition to adjunctive therapy with an SSRI, a 5-HT7 receptor antagonist may also provide useful adjunctive therapy to bright light exposure in humans. While bright light therapy has been used for several decades as a stand-alone antidepressant therapy for seasonal affective disorder (Pail et al., 2011), it has also been used in conjunction with SSRI for major depressive disorder to bolster and hasten the antidepressant properties of SSRIs since the onset of mood improvement may take several weeks and also to increase the responsiveness in those individuals who are resistant to treatment with SSRIs by themselves (Benedetti et al., 2003; Blier, 2003; Martiny, 2004; Martiny et al., 2004; Wirz-Justice et al., 2005). There is clinical evidence that bright light therapy with sertraline can be therapeutically beneficial as an adjunct therapy for depression resulting in significant reductions in the HAM-D scale of depression (Martiny et al., 2005a,b). In addition, combining bright light therapy with citalopram has been shown to accelerate the onset of the anti-depressant properties of the SSRI (Benedetti et al., 2003). Therefore, combining bright light therapy with JNJ-18038683 may enhance the antidepressant effects of the light exposure in humans. Findings from these studies help provide insight into the translational therapeutic benefits of combining a 5-HT7 receptor antagonist with additional antidepressant modalities including bright light therapy that would correct the various circadian disruptions that are commonly associated with depression.

## Conflict of interest statement

Jonathan Shelton, Sujin Yun, Curt Dvorak, Pascal Bonaventure, Timothy Lovenberg, and Christine Dugovic are full-time employees of Janssen Research and Development, L.L.C. Work performed by Dr. Fred Turek and Susan Losee Olson was part of a contract service in which they were compensated.
